# Divergent host humoral innate immune response to the smooth-to-rough adaptation of *Mycobacterium abscessus* in chronic infection

**DOI:** 10.3389/fcimb.2025.1445660

**Published:** 2025-03-18

**Authors:** Emily A. Wheeler, Patricia M. Lenhart-Pendergrass, Noel M. Rysavy, Katie R. Poch, Silvia M. Caceres, Kara M. Calhoun, Karina A. Serban, Jerry A. Nick, Kenneth C. Malcolm

**Affiliations:** ^1^ Department of Medicine, National Jewish Health, Denver, CO, United States; ^2^ Department of Pediatrics, University of Colorado, Aurora, CO, United States; ^3^ Department of Medicine University of Colorado, Aurora, CO, United States

**Keywords:** cystic fibrosis, natural antibodies, complement, neutrophils, adaptation

## Abstract

*Mycobacterium abscessus* is a nontuberculous mycobacterium emerging as a significant pathogen in individuals with chronic lung diseases, including cystic fibrosis and chronic obstructive pulmonary disease. Current therapeutics have poor efficacy. Strategies of bacterial control based on host defenses are appealing; however, antimycobacterial immunity remains poorly understood and is further complicated by the appearance of smooth and rough morphotypes, which elicit distinct host responses. We investigated the role of serum components in neutrophil-mediated clearance of *M. abscessus* morphotypes. *M. abscessus* opsonization with complement enhanced bacterial killing compared to complement-deficient opsonization. Killing of rough isolates was less reliant on complement. Complement C3 and mannose-binding lectin 2 (MBL2) were deposited on *M. abscessus* morphotypes in distinct patterns, with a greater association of MBL2 on rough *M. abscessus*. Killing was dependent on C3; however, depletion and competition experiments indicate that canonical complement activation pathways are not involved. Complement-mediated killing relied on natural IgG and IgM for smooth morphotypes and on IgG for rough morphotypes. Both morphotypes were recognized by complement receptor 3 in a carbohydrate- and calcium-dependent manner. These findings indicate a role for noncanonical C3 activation pathways for *M. abscessus* clearance by neutrophils and link smooth-to-rough adaptation to complement activation.

## Introduction

*Mycobacterium abscessus* (*Mab*) is a nontuberculous mycobacterium (NTM) and a significant pathogen in individuals with chronic pulmonary diseases such as cystic fibrosis (CF), non-CF bronchiectasis, and chronic obstructive pulmonary disease (Nick et al., 2021; Victoria et al., 2021), as well as soft tissue infections (Sepulcri et al., 2023). Subclinical infections of the lung may take years to progress to chronic pulmonary disease (Martiniano et al., 2013; Nick et al., 2022). *Mab* infections are highly resistant to most current therapies, which require prolonged multidrug regimens that often have poor tolerance and serious side effects (Daley et al., 2020). Infection occurs through environmental exposure or person-to-person transmission (Bryant et al., 2016; Honda et al., 2018), and is associated with neutrophilic inflammation.

Environmental *M. abscessus* strains exhibit a distinct smooth colony morphology when grown on mycobacterial solid media. The smooth morphotype possesses traits that may facilitate colonization, such as biofilm formation and sliding motility (Howard et al., 2006). However, it is generally less virulent and less frequently associated with disease. *In vivo*, the smooth morphotype can transition to a rough colony morphotype through a poorly understood mechanism, potentially influenced by the host environment. The rough morphotype is strongly associated with increased virulence, immunogenicity, pulmonary disease, and chronic infection (Byrd and Lyons, 1999; Sanguinetti et al., 2001; Howard et al., 2006; Rottman et al., 2007; Catherinot et al., 2009; Bernut et al., 2014; Hedin et al., 2023), though the underlying mechanisms remain unclear. These observations suggest that host factors alone are insufficient to control infection in at-risk individuals and may instead facilitate adaptation to the virulent phenotype. However, little is known about the host factors that protect healthy individuals or predispose others to *Mab* infection.

Cell envelope components of *Mab* are key determinants distinguishing the smooth and rough morphotypes. This morphological difference primarily results from the loss of glycopeptidolipid (GPL) in the smooth morphotype’s cell wall (Howard et al., 2006). The absence of GPL in the rough morphotype exposes mycobacterial-specific molecules, such as phosphatidylinositol mannosides (PIMs) and lipoproteins, leading to increased immune cell activation (Howard et al., 2006; Rhoades et al., 2009; Roux et al., 2011). Emerging insights into rough *Mab* infection are coming into focus, including enhanced cytokine production and inhibition of autophagy in macrophages (Howard et al., 2006; Rhoades et al., 2009; Roux et al., 2011; Roux et al., 2016; Kim et al., 2017). However, in neutrophils, cytokine production is similar between the morphotypes, and autophagy does not appear to be involved (Malcolm et al., 2013; Malcolm et al., 2018).

Plasma contains pattern-recognition molecules capable of binding and controlling pathogens. These opsonins converge on the activation of innate immune complement pathways. Canonical complement activation consists of the classical pathway (CP), the lectin pathway (LP), and the alternative pathway (AP). The CP is initiated by IgG and IgM binding to pathogens, leading to the binding of the C1 complex, a multiprotein protease complex consisting of C1q, C1r, and C1s. The LP is activated by binding of mannose-binding lectin-2 (MBL2) to mannose and other carbohydrates, including *N*-acetylglucosamine (GlcNAc), and is complexed with MBL2-associated serine proteases (MASPs) (Ip et al., 2009). The AP uses Factor B in an amplification mechanism to enhance the activity of C3 convertases, which are the common protease activities of all three pathways, and cleave C3 to reactive C3b and further to iC3b. Covalent association of the C3 fragments C3b and iC3b marks pathogens for recognition by the complement receptors CR1, CR3, and CR4 on neutrophils. In addition, specific antibodies can opsonize pathogens for recognition via Fc receptors, although lower-affinity nonimmune, or natural, antibodies can also act with and without complement to promote pathogen clearance (Schiller et al., 1979; Lutz et al., 1993; Schwartz et al., 2012; Lenhart-Pendergrass et al., 2023).

The role of plasma components in controlling NTM infections remains poorly understood. We recently demonstrated that neutrophil-mediated killing of the slow-growing NTM *M. avium* is enhanced by plasma complement C3 and IgM (Lenhart-Pendergrass et al., 2023). To extend these findings to rapidly growing NTM, we investigated the role of complement and immunoglobulins in neutrophil-mediated killing of *Mab* morphotypes.

## Materials and methods

### Bacterial strains, media, and culture conditions

*M. abscessus* ssp*. abscessus* (*Mab*; ATCC strain 19977) smooth and rough morphotypes were propagated from frozen aliquots in 7H9 broth supplemented with 0.5 g/l bovine albumin fraction V, 0.2 g/l dextrose, 0.3 mg/l catalase (ADC; BD Biosciences, Franklin Lakes, NJ), along with 2% glycerol and 0.05% Tween-80, at 37°C with shaking at 200 rpm for 3–4 days. Clinical isolates were obtained from the Cystic Fibrosis Foundation NTM National Resource Center at National Jewish Health. Washed cultures were sonicated using six 1-s bursts of a Fisher Sonic Dismembrator 100 at approximately 4 W output to obtain single cells and smaller aggregates, then adjusted to OD_600_ of 1.0 in PBS containing Ca^2+^ and Mg^2+^, corresponding to approximately 3 ×10^8^ CFU/ml (Pohl et al., 2020).

### Study population

Blood was drawn from healthy donors for neutrophil and plasma isolation. These studies were approved by the Biomedical Research Alliance of New York Institutional Review Board, and written informed consent was obtained from all donors. The study was conducted in accordance with the Declaration of Helsinki.

### Neutrophil isolation

Neutrophils were isolated from healthy volunteers using the plasma Percoll method, as previously described (Young et al., 2011), from blood drawn into 13 mM sodium citrate. The isolated neutrophils were washed and resuspended in Krebs–Ringer phosphate-buffered dextrose (154 mM NaCl, 5.6 mM KCl, 1.1 mM MgSO_4_, 2.2 mM CaCl_2_, 0.85 mM NaH_2_PO_4_, 2.15 mM Na_2_HPO_4_, and 0.2% dextrose). The cells were confirmed to be > 98% pure by visual inspection of cytospins.

### Plasma isolation

Citrated platelet-rich plasma from the neutrophil isolation was centrifuged at 2,000 × *g* for 15 min to obtain platelet-poor plasma and was then recalcified by adding 40 mM CaCl_2_ to allow clotting. Plasma from EDTA tubes was recalcified with 5 mM CaCl_2_ and clotted as with citrated plasma. The serum was clotted for > 1 h at room temperature, and all plasmas were isolated after centrifugation at 2,000 × *g* for 15 min. As serum tubes may contain components that inhibit complement-activating factors (Brady et al., 2014), blood was also drawn into 15 ml conical tubes without additives and clotted at room temperature for 1 h. In most cases, serum samples were recentrifuged for 5 min to remove residual red blood cells. Plasmas and sera were stored at − 20°C until ready for use. Plasma from citrated blood, designated whole plasma (WP), remained active when stored at 4°C for over 1 week. Plasmas were also incubated at 56°C for 30 min to inactivate complement, generating heat-inactivated plasma (HIP), and were likewise stored at − 20°C. Normal human serum and sera depleted of C2, C3, C4, C1q, and Factor B were purchased from Complement Technologies, Tyler, TX. The depletion of C3 and C1q had been demonstrated previously (Lenhart-Pendergrass et al., 2023).

### Immunoglobulin depletion

WP and HIP were mixed with Protein A-agarose (GoldBio, Boulder, CO), Protein G-agarose (Invitrogen, Waltham, MA), and anti-IgM-agarose (Sigma, St Louis, MO, A9935) at an approximate ratio of 1 volume of plasma to 1 volume of packed beads. The mixtures were rotated for 1 h, pelleted by centrifugation, and the depleted supernatants removed from the bead pellet.

### Opsonization reactions

Washed *Mab* (~ 1 × 10^7^) was incubated with 33% plasmas, serum, or complement-depleted sera for 20 min at 37°C (Lenhart-Pendergrass et al., 2023), then diluted in PBS to 12% plasma. The final plasma concentration was 1.2% when added to cells. In some experiments, mannan (1 mg/ml), GlcNAc (100 mM), Mg^2+^/EGTA (5 mM/10 mM), or EDTA (10 mM) was added to bacteria before the addition of WP. Opsonized *Mab* was briefly sonicated and added directly to neutrophils. For Western blotting, cells (~ 1 × 10^8^) were opsonized for 30 min in 33% plasmas and washed three times in PBS before solubilization in the SDS sample buffer. In some cases, the supernatant was retained for analysis. Plasmas and sera used were from multiple five-donor pools.

### Killing assay

Neutrophils were suspended in RPMI supplemented with 10 mM HEPES (pH 7.4) and 1%–2% pooled heat-inactivated platelet-poor plasma (complete RPMI). Sample tubes contained cells (1 × 10^6^) suspended in complete RPMI and the respective bacteria at a multiplicity of infection (MOI) of approximately 1:1 in a 0.1-ml volume. Tubes were initially centrifuged at 2,000×*g* for 1 min, and pelleted cells were incubated at 37°C for 5 min to promote bacteria–neutrophil interaction, followed by resuspension and incubation for up to 2 h, as indicated (Pohl et al., 2020). These experimental conditions promote synchronous, close contact between mycobacteria and neutrophils (Malcolm, 2018). In some experiments, 10% WP and HIP were added to neutrophils immediately before infection with nonopsonized *Mab*. Inhibitor experiments were performed by the addition of mannan (1 mg/ml), GlcNAc (100 mM), GalNAc (100 mM), and EDTA (10 mM) immediately before bacterial inoculum. Triton X-100 (0.1%) in 0.9% NaCl was added to inactivate neutrophils and facilitate mycobacterial dispersion. Following vortex mixing and serial dilutions in saline, samples were plated on 7H10 agar supplemented with OADC and incubated at 37°C. Colonies were counted 3 to 5 days after plating, and compared to colony counts at initiation of infection. Cell-free killing bacteria were incubated with WP and HIP for the opsonization reactions, added to a complete medium for the indicated times, and enumerated as described above.

### Complement receptor blockade

Neutrophils were pre-incubated for 15 min with 10 mg/ml anti-CR1 (GeneTex, Irvine, TX, GTX44217), 30 mg/ml anti-CR3 (BioLegend, San Diego, CA, clone M1/70, No. 101248), or the combination. Isotype antibody (BioLegend, No. 400644, IgG2b) was used in all experiments so that equal protein was present in all experiments.

### Western blotting

Opsonized *M. abscessus* in SDS sample buffer were heated at 95°C for 5 min and loaded onto 10% polyacrylamide gels. The gels were transferred to nitrocellulose, and *Mab*-associated proteins were detected using anti-iC3b (BioLegend, 846302), anti-C1q (Novus, Centennial, CO, NB100-64420), anti-MBL2 (Abcam, Waltham, MA, ab189856), anti-IgG-HRP (Abcam, ab97175), anti-IgA-HRP (Southern Biotech, 2050-05), and anti-IgM-HRP (Southern Biotech, Homewood, AL, 2020-05) via enhanced chemiluminescence.

### Statistics

Statistical comparisons were performed using ANOVA and *t*-tests in Excel after confirming normal distributions with the Kolmogorov–Smirnov test (https://www.statskingdom.com/kolmogorov-smirnov-test-calculator.html).

## Results

### Plasma components enhance *Mab* killing by neutrophils

**Figure 1:**
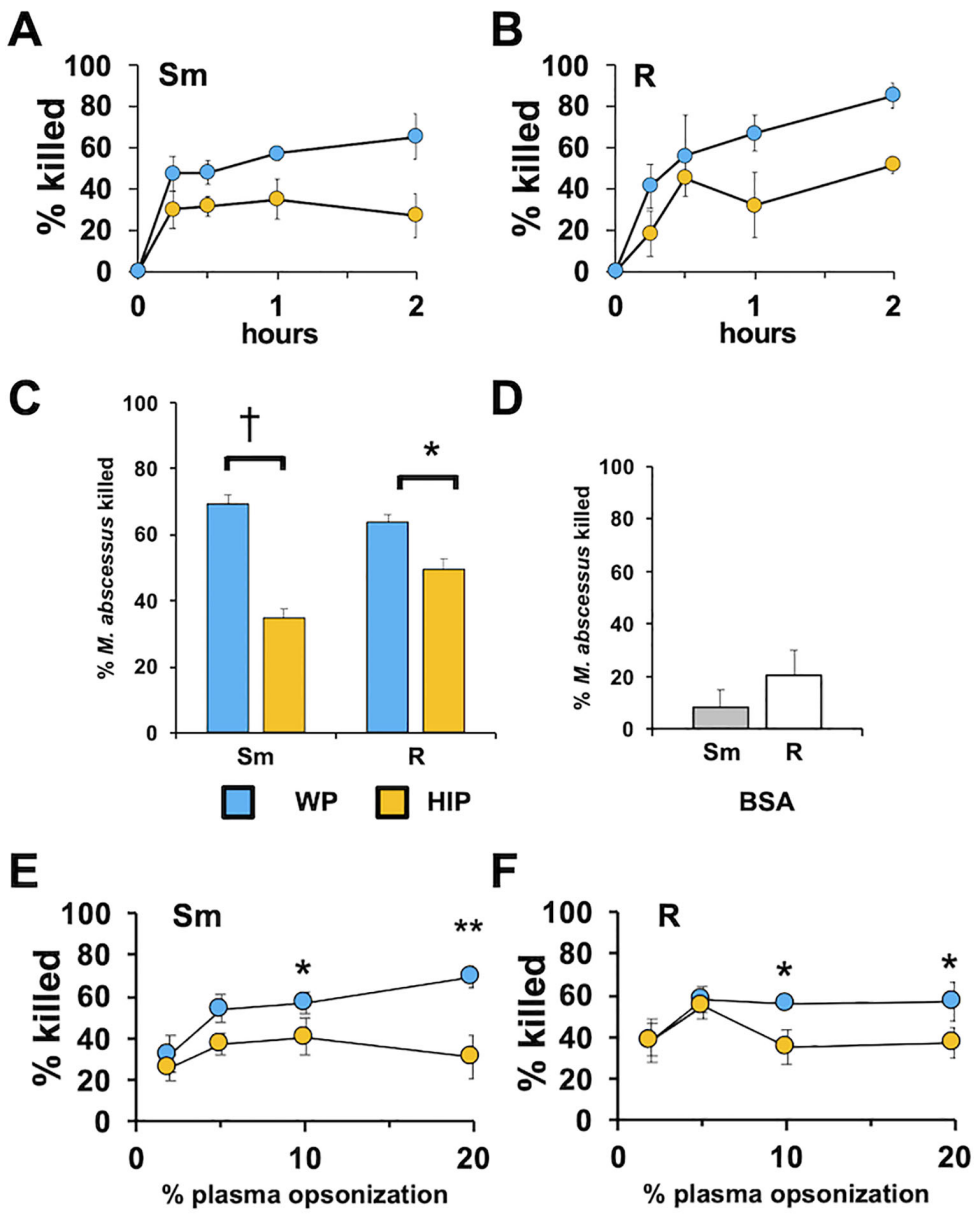
Whole plasma enhances the killing of *Mab*. **(A)** Smooth (Sm) *Mab* and **(B)** rough (R) *Mab* opsonized with whole plasma (WP; blue) or heat-inactivated plasma (HIP; orange) were added to human neutrophils at an MOI of 1 for the indicated times and killing determined; *n* = 3. **(C)** Smooth and rough *Mab* were opsonized in WP or HIP, or **(D)** incubated with BSA and killing determined after 1 h of incubation with neutrophils (*n* = 37 for Sm and R, and *n* = 4 for BSA). **(E)** Smooth and **(F)** rough *Mab* opsonized with the indicated percentage of WP or HIP and killing determined after 1 h of incubation with neutrophils (*n* = 5–6: ^*^ *p* < 0.05; **p < 0.01: ^*^ *p* < 0.05; ^†^ *p* < 0.001).

We have previously shown that in the absence of complement, *Mab* is poorly recognized or killed by human neutrophils (Malcolm et al., 2013; Malcolm et al., 2018; Pohl et al., 2020). The role of complement as an opsonin was determined by measuring *Mab* survival in the presence of human neutrophils. We compared opsonization with naïve, complement-containing WP and HIP, which is devoid of complement activity. Opsonization of both smooth and rough *Mab* with WP led to rapid and efficient killing by neutrophils (**Figures 1A, B**). Killing with WP opsonization was more robust than with HIP opsonization (**Figure 1C**). A smaller difference between killing in the presence of WP and HIP was seen for the rough morphotype of *Mab*, and the difference was significantly different between morphotypes (two-way ANOVA, *p* = 1.5 × 10^−4^). Mock opsonization with BSA resulted in less neutrophil killing than opsonization with WP or HIP (**Figure 1D**). Efficient killing occurred at plasma concentrations as low as 5% (**Figures 1E, F**). Reaction of WP or HIP in the absence of neutrophils did not affect *Mab* survival over the times used in these studies (**Supplementary Figure S1**). The heat-labile nature of plasma components suggests a role for complement opsonization in the recognition and clearance of *Mab*.

The need for pre-opsonization of *Mab*, as performed in the above experiments, was also addressed to determine if the effect of complement opsonization was influenced by the presence of neutrophils. When plasma was introduced to the neutrophils just before non-opsonized *Mab* was added, the killing of smooth *Mab* was reduced, but the killing of rough *Mab* was not changed when compared to pre-opsonized *Mab* (**Supplementary Figure S2**). Decreased killing when WP was added concurrently with nonopsonized *Mab* may reflect the activity of neutrophil-derived negative regulatory factors (e.g., CD55) or different morphotype kinetics of neutrophil recognition or of C3 activation. Drawing blood into different types of collection tubes can affect plasma components (Brady et al., 2014). Therefore, we compared the killing ability of serum and of plasmas from blood drawn into citrate, heparin, and EDTA. Citrate and EDTA WP were recalcified and clotted. Killing was similar in all conditions (**Supplementary Figure S3**), suggesting that plasma processing is not a major constraint in measuring complement function.

### Different complement deposition on smooth and rough morphotypes

**Figure 2:**
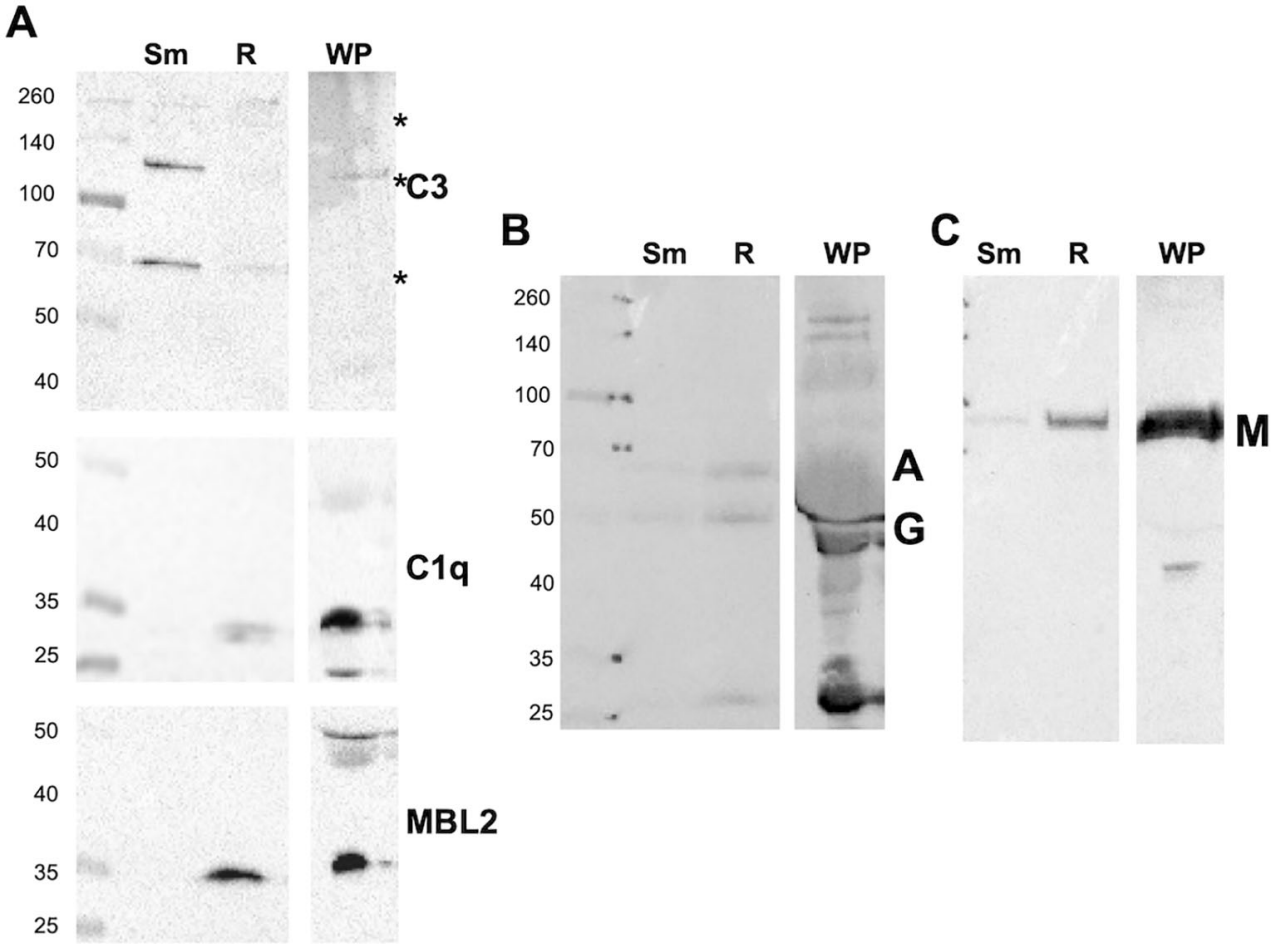
Whole plasma differently opsonizes *Mab* morphotypes. **(A)** Smooth (Sm) and rough (R) *Mab* opsonized with whole plasma were washed, proteins separated by SDS-PAGE, and deposited iC3b, C1q, and MBL2 were detected. A 1:20 dilution of WP was run as a positive control; Asterisk indicates iC3b reactivity at 70, 130, and 260 kDa. **(B)** Detection of IgG (G) and IgA (A). **(C)** Detection of IgM (M). The results are representative of over 10 separate experiments. Molecular weight markers are shown to the left of each blot.

The plasma protein components associated with *Mab* under various opsonization conditions were analyzed by immunoblotting. *Mab* was opsonized with WP, washed, and *Mab*-associated proteins extracted in an SDS sample buffer. Blots were probed with antibodies against iC3b, C1q, MBL2, IgG, and IgA (**Figures 2A–C**). C3 fragments were deposited on smooth *Mab*, whereas little MBL2 and C1q deposition was observed. The C3-positive bands represented the iC3b fragments, including those covalently bound to *Mab* moieties through the reactive ester, with major bands at 70, 130, and a group at 260 kDa. Rough *Mab* exhibited a distinct pattern of iC3b association compared to smooth *Mab*; the major 70- and 130-kDa bands were minimal, while the 260-kDa bands were present at levels similar to those in smooth *Mab*. C1q deposition was weak. MBL2 predominantly deposited on rough *Mab*. Immunoblotting revealed that the immunoglobulin classes IgG, IgA, and IgM bound both *Mab* morphotypes (**Figures 2B, C**), with no clear distinction between smooth and rough *Mab*. When HIP was used to opsonize *Mab*, no loss of IgG was detected, while C3 binding was reduced, and C3 in plasma was degraded. Surprisingly, increased C1q and MBL2 levels were observed, suggesting nonspecific binding of the inactivated proteins (**Supplementary Figure S4**). Furthermore, the deposition of complement factors on two unrelated bacterial species, *Pseudomonas aeruginosa*, and *Staphylococcus aureus*, were significantly different than that of either *Mab* morphotype (**Supplementary Figure S5**), indicating that complement activation depends on bacterial species. These data indicate morphotype-specific interaction of C3 and MBL2.

### Smooth and rough clinical isolate opsonization and killing

**Figure 3:**
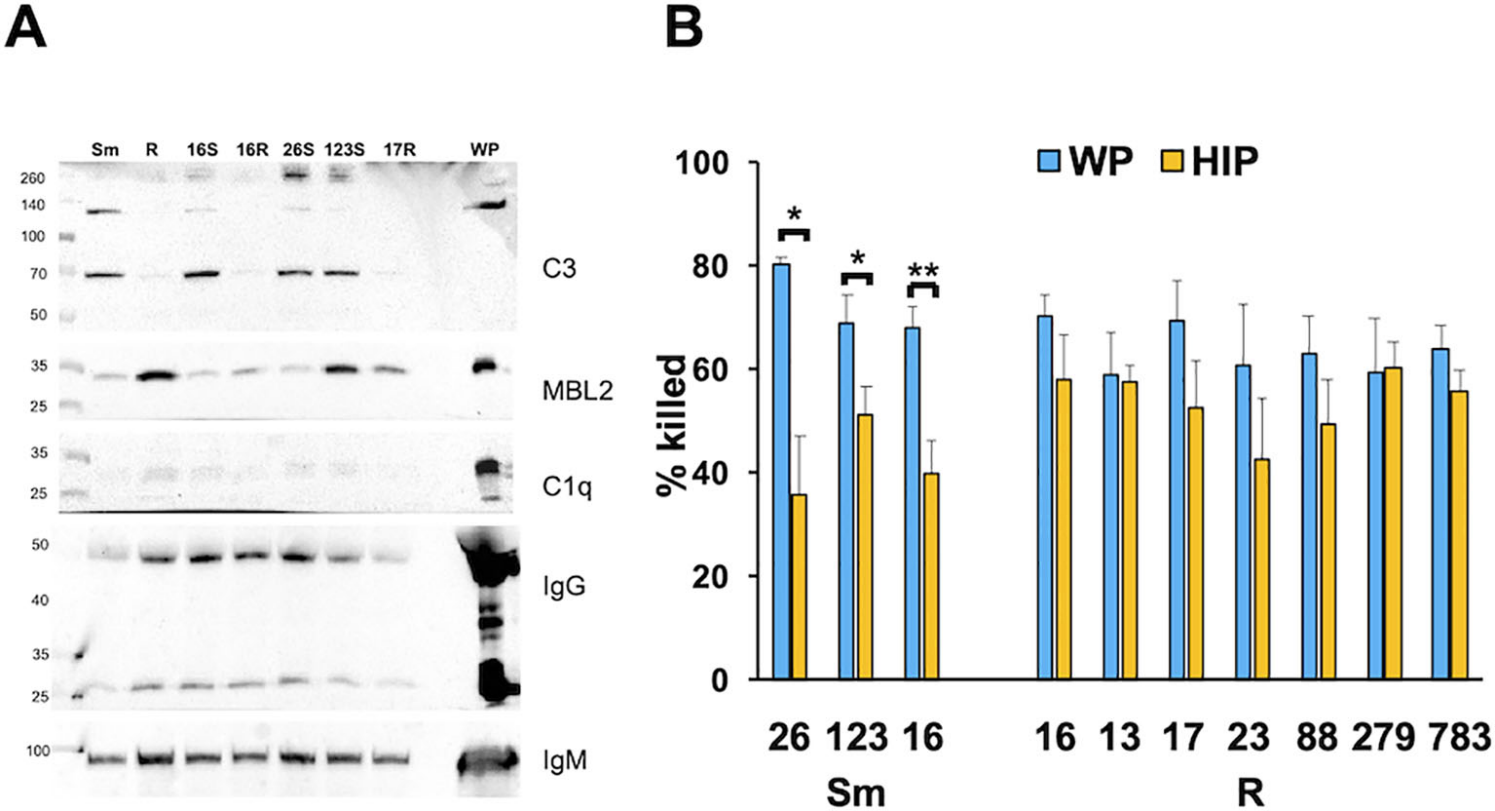
Killing of smooth *Mab* clinical isolates is more dependent on complement than rough *Mab* isolates. **(A)** Smooth (S) and rough (R) *Mab* clinical isolates (numbered by isolate) opsonized with WP were washed, proteins separated by SDS-PAGE, and deposited iC3b, C1q, MBL2, IgG, and IgM were detected. A 1:20 dilution of WP was run as a positive control. S and R designate the morphotype. ^*^ *p* < 0.05; ^**^ *p* < 0.01. **(B)** Smooth and rough *Mab* clinical isolates opsonized with WP (blue) or HIP (orange) were added to human neutrophils at an MOI of 1 for 1 h and killing determined; *n* = 4–11 per isolate.

To determine if morphotype differences between iC3b and MBL deposition were a general phenomenon, we measured complement deposition on genetically distinct CF clinical smooth and rough isolates from people with CF (Davidson et al., 2021). Like the type strains, iC3b deposition of the 70- and 130-kDa bands was predominant on the smooth strains. C1q was minimally detected. While in the two isogenic pairs (ATCC and CF016) MBL2 deposition was greater on the rough strains (**Figure 3A**), MBL2 deposition was variable overall among isolates. We observed more deposition of MBL2 on CF0123, a smooth strain, than on the genetically related, but not isogenic, rough strain CF0017-R. The effect of killing after opsonization of these clinical isolates was determined. Smooth isolates were more susceptible to heat-sensitive plasma components than rough isolates (**Figure 3B**), either individually or in composite (**Supplementary Figure S6**). These data suggest that, in general, smooth isolates are more sensitive to complement than rough isolates.

### C3 promotes neutrophil *Mab* killing

**Figure 4:**
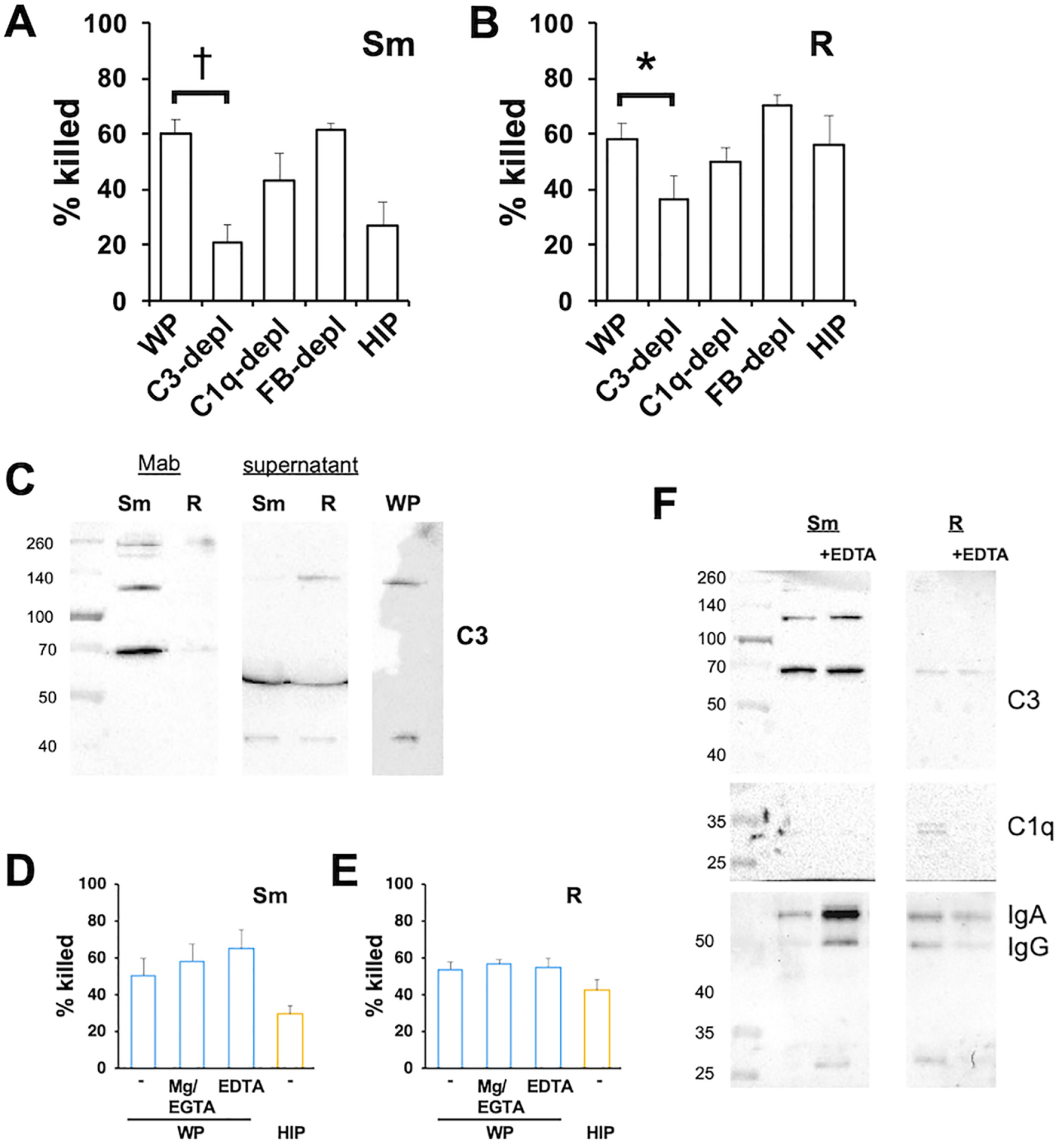
The opsonized killing of *Mab* requires C3 but is independent of CP and AP. **(A)** Smooth and **(B)** rough *Mab* opsonized with serum, sera depleted of C3, C1q, and Factor B, or HIP were added to human neutrophils at an MOI of 1 for 1 h and killing determined; *n* = 6–8. **(C)** Smooth and rough *Mab* were opsonized with WP and the supernatants retained. The washed cellular pellet and supernatant proteins were separated by SDS-PAGE, and deposited iCb3 was detected. A 1:20 dilution of WP was run as a positive control; *n* = 3–5. **(D)** Smooth and **(E)** rough *Mab* were opsonized with WP or HIP alone or in the presence of Mg^2+^/EDTA or EGTA before adding to neutrophils. Killing was determined after 1 h of incubation; *n* = 6. **(F)** Smooth and rough *Mab* were opsonized in WP or WP + EDTA, washed, and deposited iC3b, C1q, IgG, and IgA were detected; *n* = 3. ^*^ *p* < 0.05; ^†^ *p* < 0.001.

The activation of C3 is a common feature of the three major complement activation pathways. Opsonization of *Mab* with C3-depleted serum resulted in reduced killing of both morphotypes (**Figures 4A, B**), with a greater inhibition observed for smooth *Mab*. The finding that rough *Mab* killing was dependent on C3, despite minimal iC3b deposition, could be attributed to reduced C3 activation or the loss of reacted C3 from the cell surface (Bhakdi et al., 1974; Laarman et al., 2011). To investigate this, we measured residual C3 in the supernatant after the opsonization reaction. Opsonization of rough *Mab* with WP preserved C3 in the postreaction supernatant at a similar size (~ 130 kDa) as unreacted C3 in WP (**Figure 4C**), consistent with reduced C3 activation. In contrast, the postopsonization supernatant of smooth *Mab* had minimal unreacted C3, indicating efficient reaction and deposition on its surface. These data confirm a role for C3 as a smooth *Mab* opsonin while highlighting reduced C3 activation in rough *Mab*.

### Opsonization occurs in the absence of the CP and AP

To determine the role of the CP and AP on C3 activation we optimized *Mab* with serum depleted of C1q and Factor B, mediators of the CP and AP, respectively. Depletion of C1q or Factor B did not reduce killing, indicating that opsonization occurred independently of the CP and AP (**Figures 4A, B**). In addition, killing was not affected when *Mab* was opsonized with C2- or C4-depleted sera (**Supplementary Figure S7**), conditions that inhibit CP and LP activation. To confirm these findings, *Mab* was opsonized with WP in the presence of Mg^2+^/EGTA, a condition that allows only AP activation, or EDTA to chelate Ca^2+^ and Mg^2+^ and inhibit activation of CP, LP, and AP (**Figures 4D, E**). Interestingly, these conditions did not reduce *Mab* killing. Opsonization in the presence of EDTA did not affect the deposition of iC3b on *Mab* (**Figure 4F**) or the deposition of IgG or IgA. These data suggest that canonical activation of the CP, LP, and AP are not essential for WP opsonization.

### Competition of MBL2 with mannan and GlcNAc does not alter the killing of *Mab*

**Figure 5:**
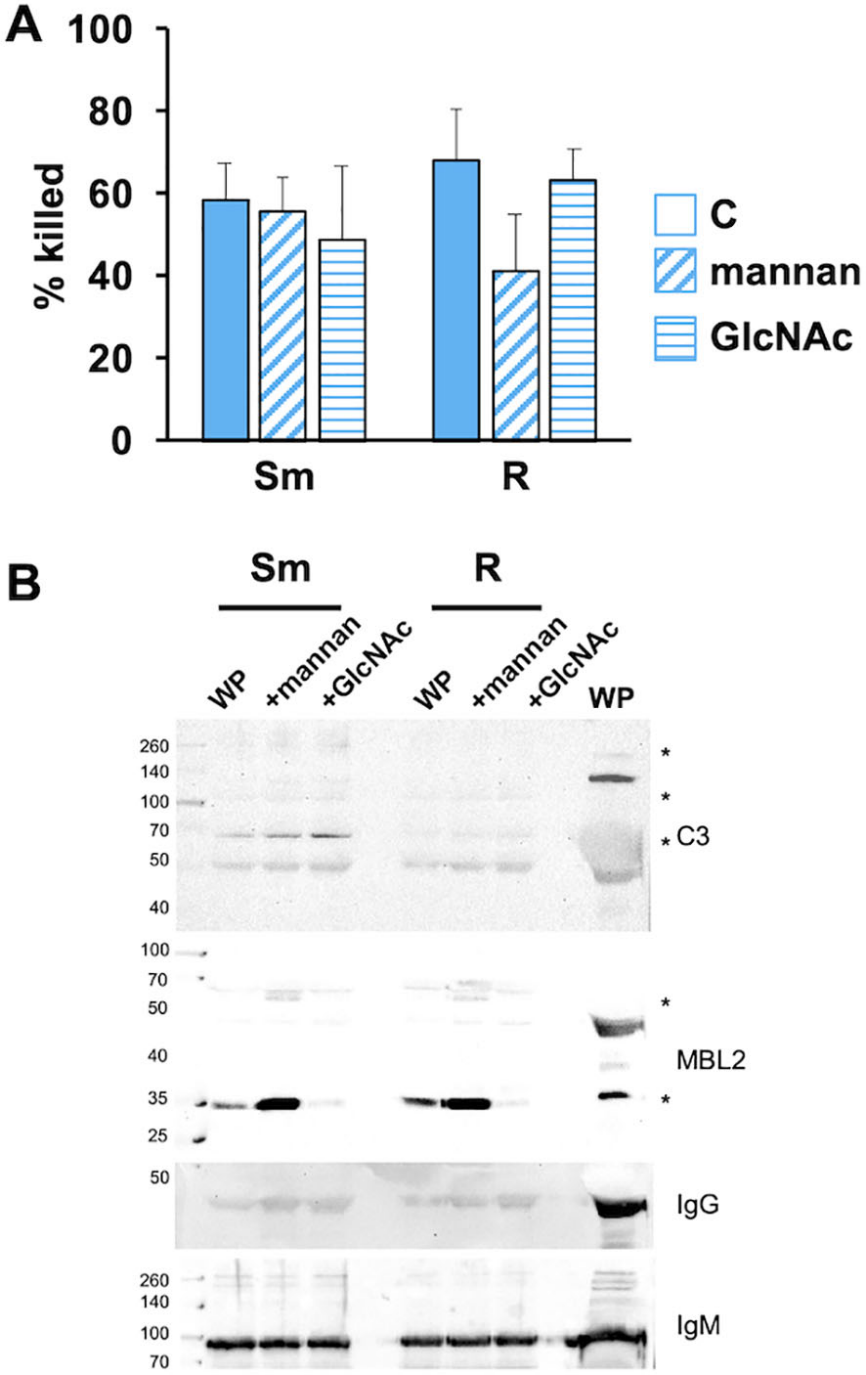
MBL2 ligands present during opsonization do not affect the killing of *Mab*. **(A)** Smooth and rough *Mab* opsonized with WP alone (solid) or in the presence of mannan (diagonal stripes; 1 mg/ml) or GlcNAc (horizontal stripes; 100 mM) were added to human neutrophils at an MOI of 1 for 1 h and killing determined; *n* = 5. **(B)** *Mab* opsonized as in **(A)** were washed, proteins separated by SDS-PAGE, and deposited iC3b, MBL2, IgG, and IgM were detected; *n* = 5. A 1:20 dilution of WP was run as a positive control. Asterisk indicates immunopositive bands.

To further address the role of MBL2 in *Mab* opsonization, we performed the opsonization reaction in the presence of competitive soluble mannan (an a-linked mannose polymer) and GlcNAc (*N*-acetyl-d-glucosamine), two agents that bind MBL2 to activate the MASP2-dependent C3-convertase. Opsonization in the presence of mannan or GlcNAc did not affect the killing of *Mab* (**Figure 5A**). In the same conditions, deposition of iC3b and MBL2 was measured. Mannan did not inhibit MBL2 or iC3b association with either morphotype (**Figure 5B**). GlcNAc reduced association with MBL2 with little effect on iC3b deposition. Neither agent affected IgG and IgM deposition. These data suggest that C3 fixation can occur independently of MBL2.

### Smooth and rough *Mab* require distinct Ig recognition to mediate opsonization

**Figure 6:**
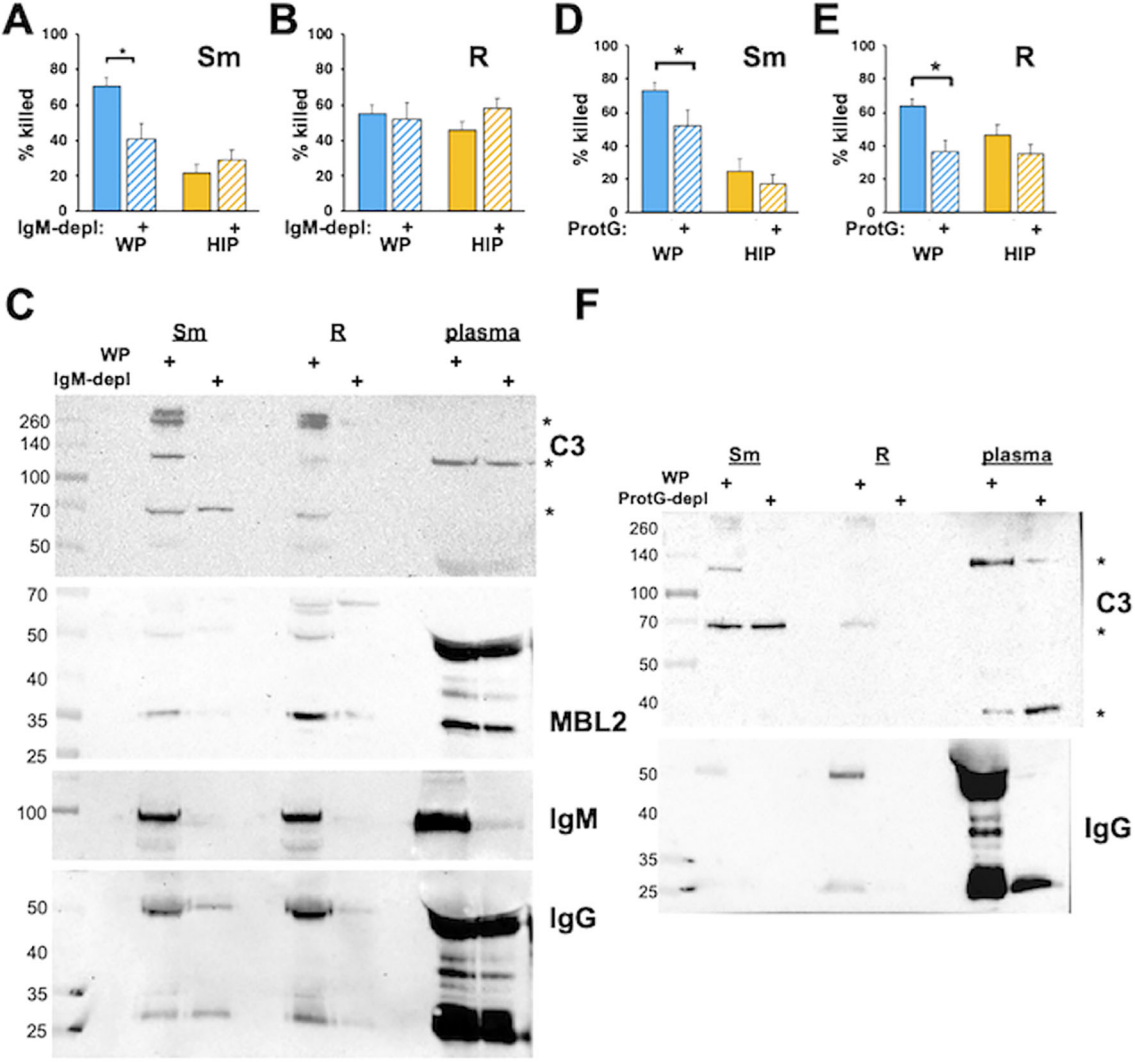
Immunoglobulins are required for the killing of *Mab* opsonized in WP. **(A)** Smooth and **(B)** rough *Mab* opsonized with WP or HIP alone (solid) or in WP or HIP depleted of IgM with anti-IgM agarose (striped bars) were added to human neutrophils at an MOI of 1 for 1 h and killing determined. **(C)** *Mab* opsonized as for **(A, B)** were washed, proteins separated by SDS-PAGE, and deposited iC3b, MBL2, IgG, and IgM were detected. A 1:20 dilution of WP or IgM-depleted WP (plasma) was run as a positive control. The asterisk indicates C3 fragments. **(D)** Smooth and **(E)** rough *Mab* opsonized with WP or HIP alone (solid) or in WP or HIP depleted of IgG with Protein G Sepharose (striped bars) were added to human neutrophils at an MOI of 1 for 1 h and killing determined; *n* = 5–10 independent experiments. **(F)** *Mab* opsonized as in **(D, E)** were washed, proteins separated by SDS-PAGE, and deposited iC3b and IgG were detected. A 1:20 dilution of WP or IgG-depleted WP (plasma) was run as a positive control. The asterisk indicates C3 fragments. *n* = 5. ^*^ *p* < 0.05.

In addition to activation of the CP, antibodies can act as opsonins to mediate phagocytosis and killing in the absence of complement. The role of immunoglobulins in the recognition and killing of *Mab* was explored using depleted plasmas. We previously observed a role of IgM in complement-dependent neutrophil *M. avium* killing (Lenhart-Pendergrass et al., 2023). To explore the role of IgM in the opsonization of *Mab*, we treated *Mab* with IgM-depleted WP. IgM-depleted WP reduced the killing of smooth, but not rough, *Mab* (**Figures 6A, B**). Depletion of IgM with anti-IgM resulted in reduced deposition of iC3b, MBL2, IgG, and IgM, although some MBL2 remained associated with rough *Mab* (**Figure 6C**). IgM depletion did not affect killing when *Mab* was opsonized with HIP, consistent with IgM lacking a direct opsonizing effect.

To understand the role of IgG, the four IgG subclasses were selectively depleted using Protein G. Opsonization in IgG-depleted WP led to reduced *Mab* killing (**Figures 6D, E**). The incomplete reduction of killing in smooth *Mab* in Protein G-depleted WP suggests that additional factors, such as IgM, may be involved. In contrast, depleting IgG from HIP had no effect on *Mab* killing, indicating that IgG opsonization does not have a direct effect in this context. Consistent with these findings, iC3b deposition was reduced in IgG-depleted serum (**Figure 6F**), as was the association of IgG with *Mab*. These data suggest that the IgG and IgM serve as opsonins for smooth *Mab*, whereas IgG alone acts as an opsonin for rough *Mab*. However, immunoglobulins are necessary but not sufficient for optimal *Mab* killing in the presence of WP. Similar results were obtained using protein A depletion, which binds IgG (except for the IgG3 subclass), IgM, and, to a lesser extent, IgA (**Supplementary Figure S8**).

### Receptor recognition of opsonized *Mab*

**Figure 7:**
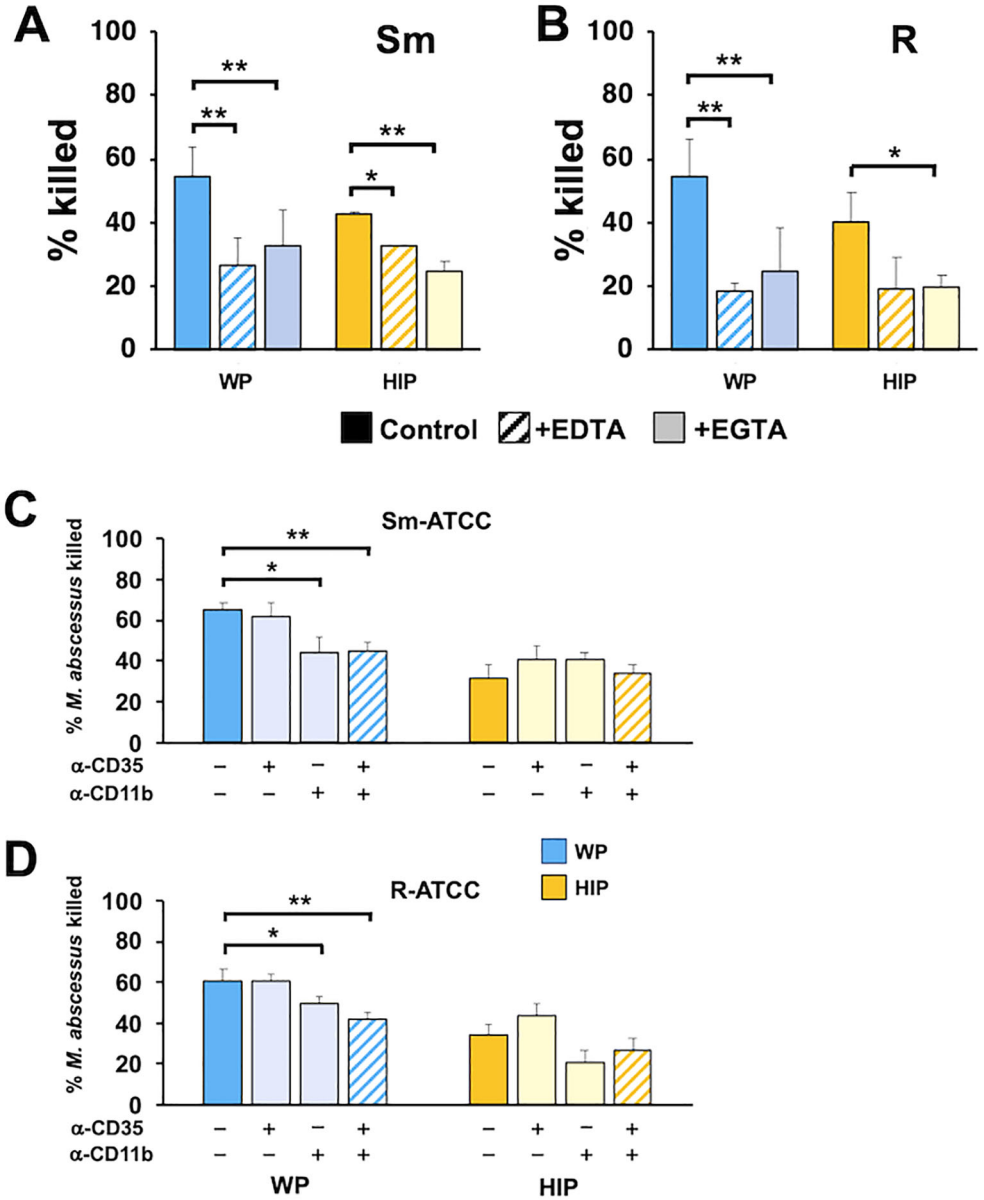
CR3/CD11b and cations play independent roles in the killing of opsonized *Mab*. **(A)** Smooth and **(B)** rough *Mab* opsonized with WP or HIP (solid) were added to human neutrophils in the presence of EDTA (striped bars) or EGTA (light shading) at an MOI of 1 for 1 h and killing determined; *n* = 4–6. **(C)** Smooth and **(D)** rough *Mab* opsonized with WP or HIP (solid) were added to human neutrophils preincubated with anti-CD35 (CR1) or anti-CD11b (CR3), as indicated, and killing determined; *n* = 5–6. ^*^ *p* < 0.05; ^**^ *p* < 0.01.

Receptors for C3 fragments include the integrin family of adhesion receptors, which require divalent cations for efficient binding (Ueda et al., 1994). Reducing Ca^2+^ levels using EGTA and depleting both Ca^2+^ and Mg^2+^ with EDTA during the killing assay led to decreased killing of *Mab* opsonized with WP and HIP (**Figures 7A, B**), suggesting that calcium-dependent recognition plays a crucial role in both complement- and noncomplement-mediated opsonization by neutrophils. To specifically examine the role of neutrophil complement receptors, we used blocking antibodies against CR1 (CD35), which binds C3b, and CR3 (CD11b/CD18), which binds iC3b. Anti-CD11b partially inhibited killing (**Figures 7C, D**), whereas anti-CD35 had no effect. The combination of anti-CD35 and anti-CD11 (Schwartz et al., 2012) produced a similar effect to anti-CD11b alone. However, inhibition by anti-CD11b did not reduce killing to the same extent observed for HIP-opsonized *Mab*. These data indicate that CR3 plays a role in recognizing WP-opsonized with *Mab*, and that divalent cations are important for both complement-dependent and complement-independent recognition by neutrophils.

### Carbohydrates block *Mab* killing

**Figure 8:**
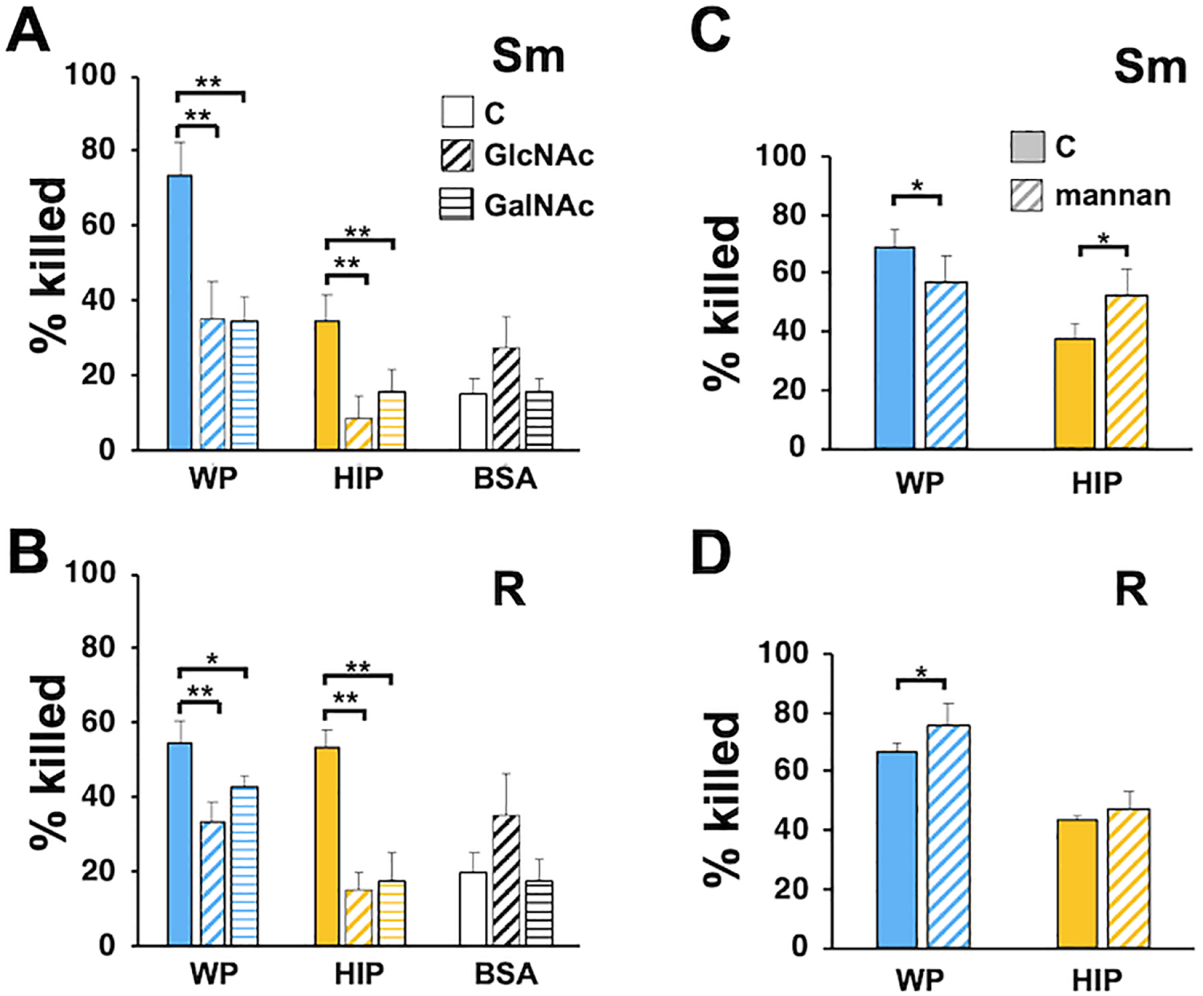
Inhibition of *Mab* killing by *N*-acetylated sugars. **(A)** Smooth and **(B)** rough *Mab* opsonized with WP or HIP alone (solid) or incubated with BSA were added to human neutrophils in the presence of GlcNAc (diagonal stripes; 100 mM) or GalNAc (horizontal stripes; 100 mM) for 1 h and killing determined; *n* = 5. **(C)** Smooth and **(D)** rough *Mab* opsonized with WP or HIP alone (solid) were added to human neutrophils in the presence of mannan (diagonal stripes; 1 mg/ml) for 1 h and killing determined; *n* = 6; ^*^ *p* < 0.05; ^**^ *p* < 0.01.

To further define complement recognition receptors, we explored the ability of free carbohydrates that mimic surface moieties to affect *Mab* killing. GlcNAc, GalNAc (*N*-acetyl-d-galactosamine), and mannose are sugars found on mycobacterial cell walls (Abrahams and Besra, 2021) and on host cells and can interact with host and pathogen receptors (Beuth et al., 1988; Kolbe et al., 2019; Sung et al., 2020). Incubation of neutrophils with either GlcNAc or GalNAc reduced the killing of *Mab* opsonized with either WP or HIP (**Figures 8A, B**). They had no effect on the minimal killing of *Mab* mock-opsonized with BSA. Incubation with mannan did not consistently affect *Mab* killing (**Figures 8C, D**); minimal inhibition of smooth *Mab* opsonized with WP was seen, but a small, enhanced killing of HIP-opsonized smooth *Mab* and WP-opsonized rough *Mab* was also observed. Therefore, recognition of *N*-acetylated sugars, but not mannose, is important for neutrophil killing of *Mab*, although these carbohydrate interactions likely involve both complement-dependent and complement-independent mechanisms.

## Discussion

Host mechanisms for containing NTM infections remain poorly understood. While innate immune cells play a role in infection control, the precise mechanisms of clearance have not been thoroughly addressed. Furthermore, most studies have focused on the interaction of myeloid cells and NTM in isolation, without considering other host-derived factors. The uptake and killing of the slow-growing NTM *M. avium* by neutrophils is enhanced in a complement- and IgM-dependent manner (Hartmann et al., 2001; Lenhart-Pendergrass et al., 2023). However, the role of complement in controlling rapidly growing NTM has not yet been explored. Here, we demonstrate for the first time that rapidly growing NYM undergoes antibody-dependent complement opsonization.

Our findings are notable in that the adapted, rough form has developed mechanisms to evade two different innate immune defenses—C3 and IgM opsonization—resulting in MBL2 and IgG specificity. This suggests that C3 and IgM serve as in-host selective pressures for adaptation in the diseased lung. However, this adaptation comes at a cost, as it promotes MBL2 opsonization while preserving the effects of IgG. The observations that C3 facilitates *Mab* clearance and that more severe *Mab* pulmonary disease is linked to a slow conversion to the rough morphotype suggest that a delicate balance exists between adaptation and immune control of the two morphotypes. The emergence of specific morphotypes may depend on microenvironments with varying growth conditions, complement availability, or Ig class repertoires. For instance, vascular leakage may enrich complement levels in an injured lung compared to a healthy lung (Fidler et al., 2009). Killing of both morphotypes was reduced in C3-depleted serum, suggesting that even the minimal C3 deposition, as indicated by the 260-kDa bands on rough *Mab*, is sufficient to drive some level of killing. These data also imply that plasma-derived complement can effectively limit systemic infection.

Differences in complement activation and function between the morphotypes are further highlighted by clinical isolates. Killing of smooth *Mab* isolates was more dependent on heat-sensitive plasma components than that of rough *Mab* isolates. These data support the idea that the smooth morphotype may establish a foothold for sustained infection in infection sites that are limited or deficient in complement factors (Malcolm et al., 2013; Malcolm et al., 2018). The transition to the more virulent rough morphotype may occur under prolonged selective pressure in the presence of complement, possibly due to vascular leakage. Recent reports indicate reduced complement levels in the CF lung (Hayes et al., 2022), but not blood (Lenhart-Pendergrass et al., 2023), further supporting this idea. Whether MBL2 deposition on rough *Mab* is associated with virulence and disease progression remains to be studied. However, tissue injury by excessive complement activation is possible.

The dependence of complement activation on antibodies was unexpected. The plasma donors were healthy, and we previously demonstrated minimal anti-*Mab*-specific antibodies in healthy individuals (Malcolm et al., 2022). These findings implicate natural antibodies in complement activation, as previously reported (Schiller et al., 1979; Lutz et al., 1993; Schwartz et al., 2012; Khandelwal et al., 2018; Lenhart-Pendergrass et al., 2023). The antibody specificities required for full opsonization of the morphotypes may reflect changes in cell surface components. The loss of glycopeptidolipids (GPL)—a hallmark of rough *Mab*—is accompanied by exposure to PIMs (Rhoades et al., 2009) and overexpression of lipoproteins (Roux et al., 2011). These exposed molecules may serve as novel epitopes for nonimmune IgG. Other differences in cell surface composition may also play a role (Dubois et al., 2018; Daher et al., 2020; Palcekova et al., 2020). Additionally, differences in host Fc receptor utilization or activation may contribute to specificity. The interaction of CR3 (CD11b/CD18) and FcgRIII suggests one possible mechanism (Zhou et al., 1993; Galon et al., 1996), but the exact mechanism of antibody-complement interaction remains unclear and is expected to be complex.

Surprisingly, C3 activation by smooth *Mab* is IgG- and IgM-dependent but appears to be independent of the CP, as evidenced by the lack of killing inhibition after opsonization with C1q-depleted serum, minimal C1q deposition on *Mab*, and the inability of EDTA to block opsonization. Similarly, the AP does not seem to play a major role, as killing and iC3b deposition are not inhibited by EDTA, and Factor B-depleted serum does not reduce killing. A similar CP- and AP-independent mechanism has been observed for the killing of *M. avium* (Lenhart-Pendergrass et al., 2023). Reduced MBL2 association with smooth *Mab*, along with the lack effect of EDTA effects on iC3b deposition or killing, does not support a role for the LP. Furthermore, GlcNAc reduced MBL2 deposition, it did not affect killing. These data suggest that canonical complement activation is not a major driver of smooth *Mab* killing.

Opsonization of rough *Mab* also does not appear to involve CP and AP. However, the role of LP in the opsonization of rough *Mab* remains unclear. Rough *Mab* preferentially associates with MBL2; however, its killing was not blocked by EDTA, Mg^2+^/EGTA, mannan, or GlcNAc. Additionally, MBL2 deposition in the presence of these agents did not correlate with killing—reducing divalent cations or adding mannan did not affect MBL2 deposition, while GlcNAc significantly reduced MBL2 deposition. Killing of rough *Mab* was also unaffected by opsonization with C2- or C4-depleted sera, conditions that prevent MASP2-activated C3 convertase activity. These data suggest that *Mab* killing involves pathways distinct from canonical C3 activation. This discrepancy may be explained by direct MBL2 or C3 opsonization (Kuhlman et al., 1989; Polotsky et al., 1997; Neth et al., 2000), a mechanism previously demonstrated for *M. avium*. The surface exposure of PIMs or mannose-capped lipoarabinomannan in rough *Mab* may underlie the association of MBL2 with rough *Mab*. In addition, MBL2 can activate C3 in the absence of C2/C4 activation, through a process known as “C2/C4 bypass” (Dumestre-Perard et al., 2008). However, the interaction between C3 and MBL2 on rough *Mab* remains unresolved. Notably, C3 deposition on rough *Mab*, while reduced, is still detectable and functionally significant—particularly in high molecular weight conjugates. This observation explains the partial but not complete killing of rough *Mab* in C3-depleted serum and when CR3 is blocked.

Noncanonical plasma opsonins and complement activators could explain the morphotype-specific profiles of C3 activation. Alternative factors may include members of the pentraxin family (pentraxin 3, C-reactive protein, and serum amyloid P) or ficolins (FCNs), all of which are pattern-recognition receptors associated with complement activation (Haapasalo and Meri, 2019). Preliminary experiments showed little to no deposition of FCN1, FCN2, FCN3, or pentraxin 3 on *Mab* (data not shown); however, these results may have been limited by poor antibody recognition. In addition, because ficolin binding is Ca^2+^-dependent (Zacho et al., 2012) and EDTA did not reduce iC3b deposition or killing, it is unlikely that ficolins are involved. Further studies are warranted to clarify alternative pathways for C3 deposition.

Evidence supports the recognition of WP-opsonized *Mab* by CR3, an integrin receptor that plays a major role in the binding and phagocytosis of iC3b-opsonized pathogens (Dustin, 2016). Antibody blockade of CR3 reduced *Mab* killing, and the inhibition of killing by EGTA and EDTA further supports the role of integrins. The finding that blocking CR3 antibodies does not affect HIP-opsonized *Mab* suggests that CR3 does not recognize intrinsic *Mab* surface molecules. CR3 binds GlcNAc, which may interfere with the interaction of CD11b and FcgRIII (Galon et al., 1996). However, the profound inhibition of killing by GlcNAc and GalNAc in both WP- and HIP-opsonized *Mab*, when present with neutrophils, indicates that noncomplement lectins are also involved. The greater inhibition by acetylated sugars may also reflect the ability of bacterial lectins to recognize host GlcNAc (Beuth et al., 1988; Kolbe et al., 2019). The killing was not blocked by the neutralizing TLR2 antibody TL2.1 (not shown). Additionally, nonreceptor mycobacterial recognition has previously been described (Nakayama et al., 2016). These data suggest that both CR3 and noncomplement-dependent receptors play key roles in *Mab* recognition.

This study has limitations. We were unable to completely define the complement activation pathways or the recognition receptors. The deposition of C3 on *Mab* in the absence of CP, AP, or LP is unusual. Complement activation is activated by both positive and negative regulators, which were not explored. Our studies focused on the role of nonimmune plasma. While we have previously demonstrated the presence of anti-*Mab* IgG in NTM-infected individuals with CF (Malcolm et al., 2022), the impact of specific immunity on opsonization and killing remains a subject of future investigation. Finally, these studies focus on neutrophils; the role of complement in other immune cells and its *in vivo* effects require further exploration.

In summary, we have identified C3-dependent killing of *Mab* by neutrophils, which requires distinct opsonization patterns for the smooth and rough morphotypes. Opsonization of *Mab* involves noncanonical complement activation that depends on restricted natural antibody classes. Morphotype-specific shifts in complement and antibody use suggest that complement activity may be a disease risk factor and implicate complement in *Mab* adaptation during chronic infection.

## Data Availability

The datasets presented in this article are not readily available. Requests to access the datasets should be directed to malcolmk@njhealth.org.
