# Supplementary material for: Divergent host humoral innate immune response to the smooth-to-rough adaptation of Mycobacterium abscessus in chronic infection
Source: Front Cell Infect Microbiol. 2025 Mar 18;15:1445660. doi: 10.3389/fcimb.2025.1445660 (PMC11959001; doi:10.3389/fcimb.2025.1445660)
Supplement: Supplementary file 1 [file DataSheet1.pdf]

Suppl Fig 1

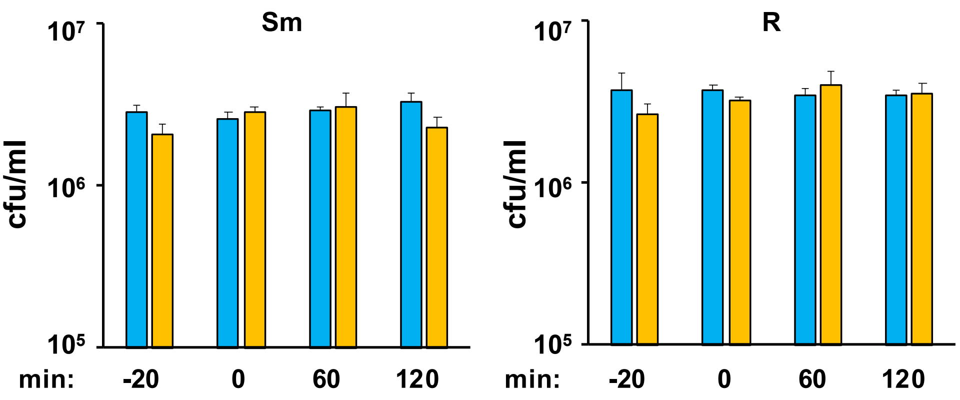

Supplemental Fig 1. Mab survives opsonization in the absence of neutrophils. Smooth (Sm) and rough (R) Mab was opsonized with WP (blue) or HIP (orange) alone and incubated for the indicated times. The -20 and 0 times are the beginning and end of the 20 min opsonization reaction, respectively ; n= 3. Mab were plated and counted to determine survival.

Suppl Fig 2

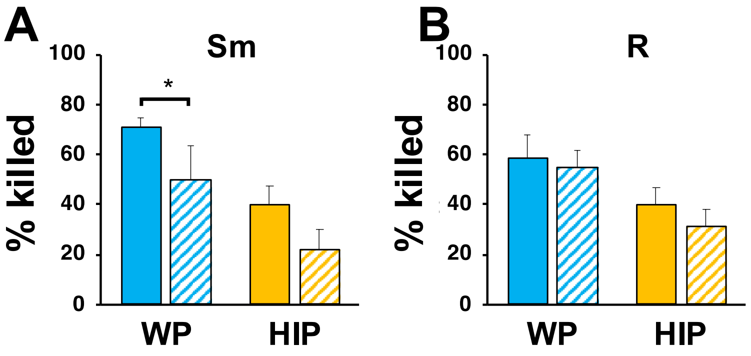

Supplemental Fig 2. Effect of plasma components in the presence of neutrophils. **A**, Smooth and **B**, rough Mab were pre-opsonized in WP or HIP (closed bars), or non-opsonized Mab was added to neutrophils after addition of 10% WP or HIP (striped bars) and killing determined after 1 hr incubation with neutrophils; n=5.

Suppl Fig 3

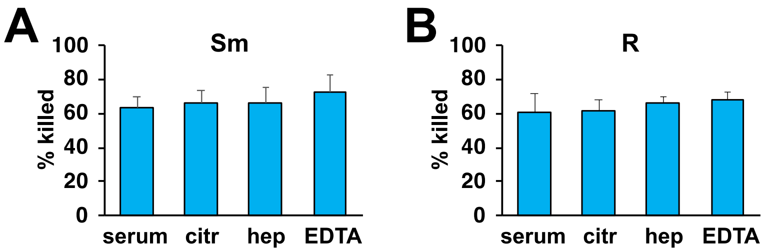

Supplemental Fig 3. Serum and plasma sources are similarly effective in supporting Mab killing. **A**, Smooth Mab and **B**, rough Mab opsonized with serum, or whole plasmas from blood drawn into citrate, heparin, or EDTA were added to human neutrophils at an MOI of 1 for 1 hr and killing determined; n=4.

Suppl Fig 4

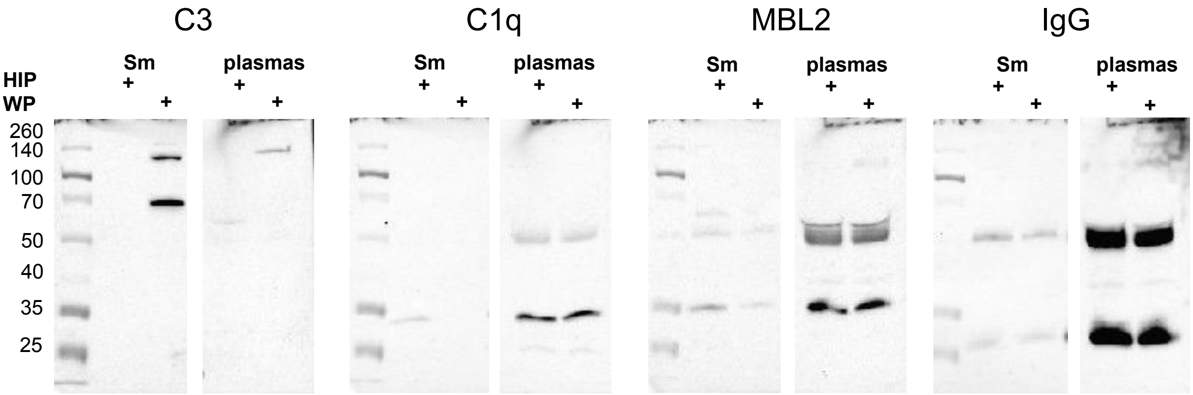

Supplemental Fig 4. Reduced C3 deposition on Smooth Mab when opsonizing with HIP. Western blot of smooth Mab opsonized with WP or HIP. Proteins were separated by SDS-PAGE, and deposited iC3b, C1q, MBL2, and IgG were detected. Complement components and IgG were also detected in plasma samples. Note the degradation of full-length C3 in HIP.

Suppl Fig 5

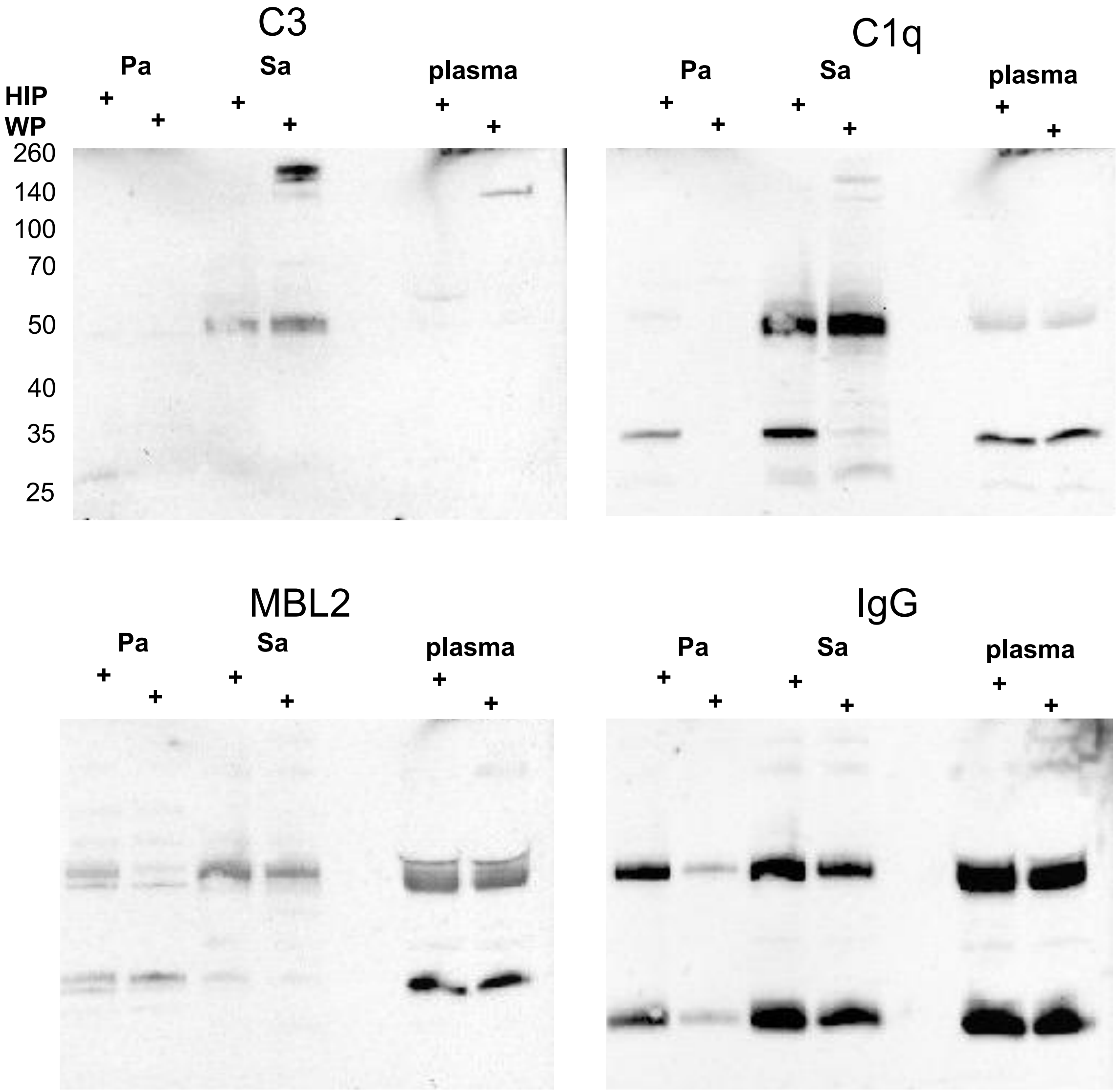

Supplemental Fig 5. Differential complement deposition on Pseudomonas aeruginosa (Pa) and Staphylococcus aureus (Sa) by WP and HIP. Western blot of Pa and Sa opsonized with WP or HIP. Proteins were separated by SDS-PAGE, and deposited iC3b, C1q, MBL2, and IgG were detected. Complement components and IgG were also detected in plasma samples.

Suppl Fig 6

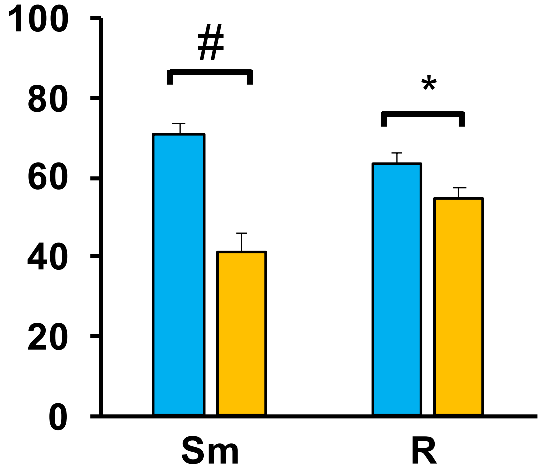

Supplemental Fig 6. Composite data of killing of smooth and rough Mab clinical isolates. Data from experiments in Fig 3 were combined to observe the general effect on killing of Mab morphotypes; n = 21 smooth and 42 rough independent experiments from 3 smooth isolates and 7 rough isolates. WP (blue) and HIP (orange); *, $P<0.05$; #$P<10^{-5}$.

Suppl Fig 7

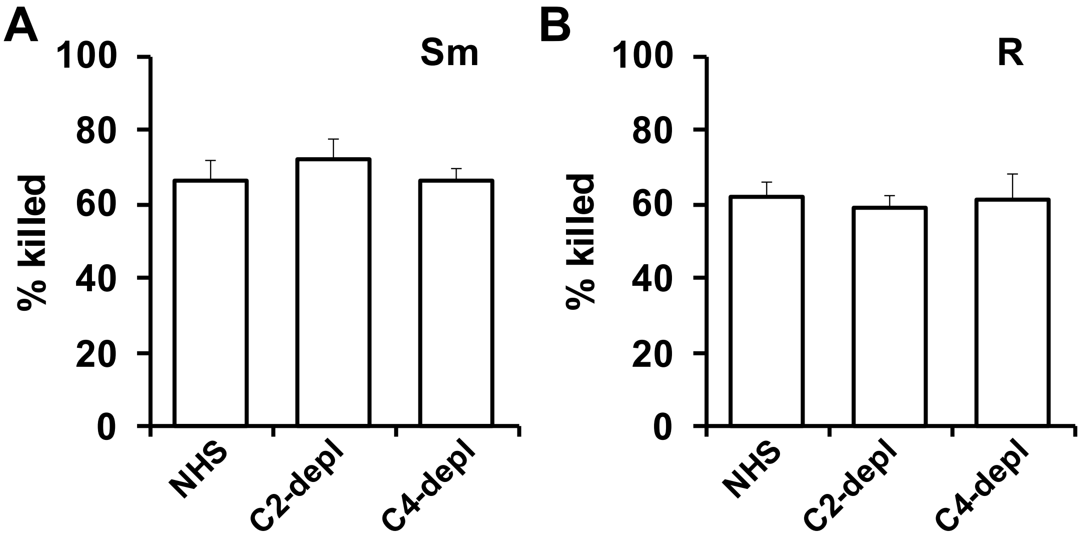

Supplemental Fig 7. Opsonized killing of Mab is independent of C2 and C4. **A**, Smooth Mab and **B**, rough Mab, opsonized with normal human serum or sera depleted of C2 and C4 were added to human neutrophils at an MOI of 1 for 1 hr and killing determined ; n = 6-8.

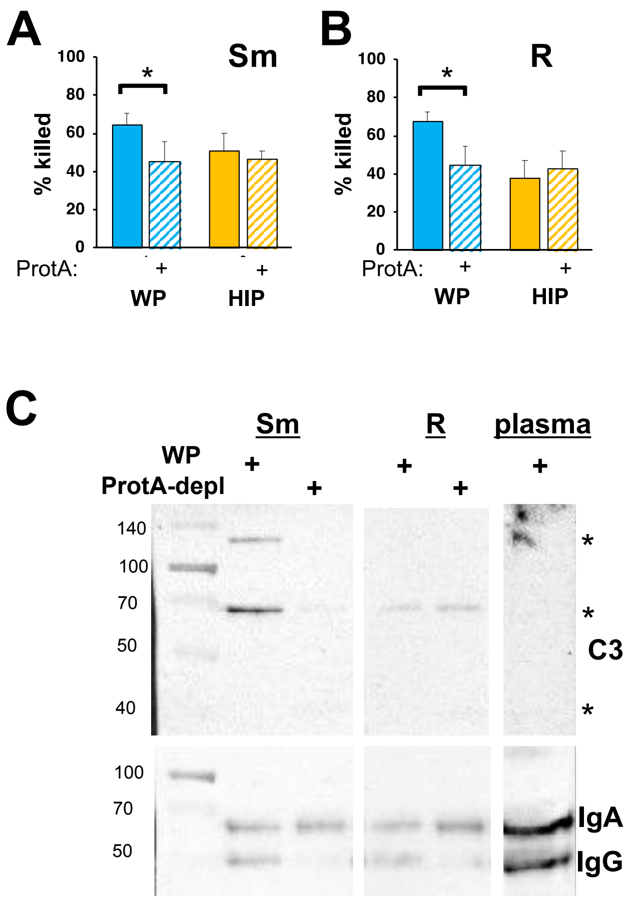

Supplemental Fig 8. Reduced neutrophil killing of Mab after opsonization with Protein A-depleted plasmas. **A**, Smooth and **B**, rough Mab opsonized with WP or HIP alone (solid) or in WP or HIP depleted of IgG and IgM with Protein A agarose (striped bars) were added to human neutrophils at an MOI of 1 for 1 hr and killing determined; n= 6. **C**, Mab opsonized in WP as in A were washed, proteins separated by SDS-PAGE, and deposited iC3b, IgG, and IgA were detected. A 1:20 dilution of WP was run as a positive control. *, $P<0.05$.
